# Species Delimitation and Morphological Divergence in the Scorpion *Centruroides vittatus* (Say, 1821): Insights from Phylogeography

**DOI:** 10.1371/journal.pone.0068282

**Published:** 2013-07-05

**Authors:** Tsunemi Yamashita, Douglas D. Rhoads

**Affiliations:** 1 Department of Biological Sciences, Arkansas Tech University, Russellville, Arkansas, United States of America; 2 Department of Biological Sciences, 601 SCEN, The University of Arkansas, Fayetteville, Arkansas, United States of America; George Washington University, United States of America

## Abstract

Scorpion systematics and taxonomy have recently shown a need for revision, partially due to insights from molecular techniques. Scorpion taxonomy has been difficult with morphological characters as disagreement exists among researchers with character choice for adequate species delimitation in taxonomic studies. Within the family Buthidae, species identification and delimitation is particularly difficult due to the morphological similarity among species and extensive intraspecific morphological diversity. The genus *Centruroides* in the western hemisphere is a prime example of the difficulty in untangling the taxonomic complexity within buthid scorpions. In this paper, we present phylogeographic, Ecological Niche Modeling, and morphometric analyses to further understand how population diversification may have produced morphological diversity in *Centruroides vittatus* (Say, 1821). We show that *C. vittatus* populations in the Big Bend and Trans-Pecos region of Texas, USA are phylogeographically distinct and may predate the Last Glacial Maximum (LGM). In addition, we suggest the extended isolation of Big Bend region populations may have created the *C. vittatus* variant once known as *C. pantheriensis*.

## Introduction

Scorpions are an ancient and widespread arthropod order well known for their medical importance as venomous arachnids [1]. Less known is their importance as model organisms for ecological research comprising key components of desert food webs [2]. These desert scorpion species have also been shown to exhibit ecomorphological specialization upon specifc habitats and possess morphological adaptations to unique edaphic substrates such as sand [2]–[4]. These edaphic specialist species illustrate the role of environmental effects upon scorpion morphological divergence and speciation. Other orogenic features such as mountain ranges can also produce profound effects upon scorpion species diversification and, until recently, these isolation effects were not fully understood. For example, geographic heterogeneity has been shown to differentiate the singular *Buthus occitanus* into several cryptic lineages [5]. Other recent studies have shown the importance of mountainous terrain and riverine barriers on the diversification of scorpions [6]–[9]. These recent studies also illustrate the impact of molecular taxonomy in revealing patterns of diversity unrepresented through traditional morphological analyses.

The current appreciation of scorpion diversity underscores the need for multiple lines of evidence to establish species delineation in scorpions. The importance of accurate species delineation is of paramount importance in the medically important Buthid family as accurate identification is needed for medical treatment of envenomation. One example of taxonomic ambiguity is illustrated in the Buthid genus *Hottentotta*. Sequence analysis of this genus, with the mtDNA Cytochrome Oxidase I, has challenged its current taxonomy as the COI sequences suggest a paraphyletic relationship in this genus’ mtDNA [10].

The Buthid genus *Centruroides*, widely distributed in the Western Hemisphere and well known for its medical importance to humans, has also confounded scorpion taxonomists [1], [11], [12]. Species of this genus can exhibit considerable intraspecific morphological variability leading to taxonomic confusion [13], [14]. For example, *Centruroides exilicauda* (Wood, 1863) in Baja California Sur, Mexico not only exhibits dramatic size variation from small, mainland individuals to gigantism on offshore islands near La Paz, but color variation ranging from pale forms north of La Paz to striped populations south of La Paz [13], [15]. An example of taxonomic uncertainty within *Centruroides*, occurred when *C. sculpturatus* (Sonora, Mexico and Arizona, USA) was synonymized into *C. exilicauda* (Baja California) [13], [16]. Valdez-Cruz et al. [17] unravelled this taxonomic mess with venom and molecular data, separating *C. sculpturatus* from *C. exilicauda* once again.

In the United States of America, not only has taxonomic uncertainty existed in the western *Centruroides* species, but also in the eastern *Centruroides vittatus* (Say, 1821*)*. This species was separated into three different species (*C. vittatus, C. chisosarius,* & *C. pantheriensis*) after morphological investigation, but later synonymised as *C. vittatus* [18]–[20]. Both *C. chisosarius* (Gertsch, [18]) and *C. pantheriensis* (Stahnke, [19]) were originally described from the Big Bend region of Texas. *C. chisosarius* was recognized as exhibiting dark spots on its carapace with darker pigmentation on the tergites [20]. *C. pantheriensis* was the most morphologically distinct, with a pale color and resembling the more medically significant Arizona *C. sculpturatus* [19]. *C. vittatus*, in the eastern portion of its geographic range exhibits dark coloration with dorsal metasomal stripes [20], [21].

Due to the confusing taxonomic history and the documented morphological diversity within this species, employing a phylogeographic approach to studying *C. vittatus* lineages should illustrate how pieces of evidence from diverse datasets can assist with species delimitation. We conducted phylogeographic, morphometric, and Ecological Niche Modelling analysis (ENM) of *Centruroides vittatus* to further understand the evolution of the *C. pantheriensis* variant within *C. vittatus*. Within our phylogeographic analyses, we conducted additional tests of several alternative tree topologies (hypotheses generated through initial review of results) based upon significance of Bayes factors [22]–[24].

As stated above, *C. chisosarius,* and *C. pantheriensis* are currently recognized as color variants of *C. vittatus*; yet, several questions remain regarding these variants. First, both were initially described and regarded as inhabitants of the Texas Big Bend region. Does *C. vittatus* from this area represent a unique phylogeographic clade? That is, do all color variants represent morphological variants within a distinct regional clade? The placement of the *C. pantheriensis* variant within such a clade appears possible as this variant appears to be restricted to the Big Bend region. In addition, is the pale color variant (*C. pantheriensis*) associated with a clade exhibiting deeper divergence (earlier evolutionary separation) when compared to other phylogeographic clades within the species? The Big Bend region possesses higher scorpion species diversity than other Texas regions [25] and may be the result of a long evolutionary history within the region. Furthermore, are there additional morphometric characters that distinguish the pale *C. pantheriensis* color variant from other *C. vittatus* populations? Is the pale form associated with a specific environmental habitat within *C. vittatus’* geographic range? Investigating these questions from a phylogeographic perspective can provide insight into the evolution of morphological variation and species delimitation in this genus.

## Materials and Methods

### Study Species

**Figure 1:**
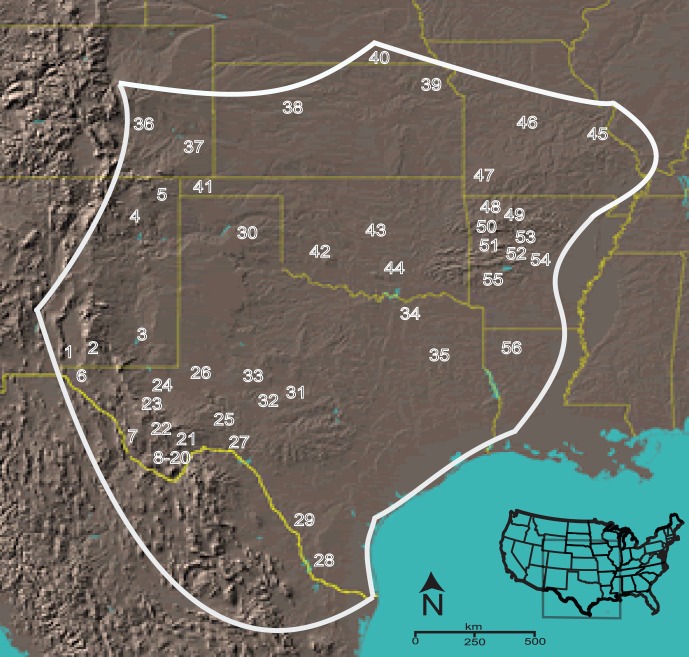
*Centruroides vittatus* collection sites and approximate geographic range boundaries. Each number corresponds to a collection site described in Table S1 with numbers 8 to 20 representing populations in the Big Bend region of Texas, USA. The inset map shows the approximate map location within the USA.

*Centruroides vittatus* encompasses a large geographic range within the U.S.A. that includes Texas, Oklahoma, New Mexico, Colorado, Kansas, Nebraska, Missouri, Arkansas, and Louisiana as well as sections of several states in the United Mexican States (Figure 1). This species, as do most *Centruroides* species, comprise errant or wandering scorpions that do not construct a burrow and commonly invade human habitations [26], [21]. Throughout its geographic range, *C. vittatus* is commonly found in diverse ecological habitats, but in populations across the northern and eastern geographic distributions it appears to prefer dry, rocky south facing slopes or glade areas. Human introduction of this scorpion appears to also have created additional populations outside its known geographic range [21].

### Field Collections

*C. vittatus* individuals were field collected throughout its US geographic range or obtained from collections through other personnel (Figure 1 & Table S1). All necessary permits were obtained for the described field studies. After field collection, all samples were stored in 95% alcohol at −20°C. GPS coordinates were identified with a hand-held unit (Magellan GPS 315, Santa Clara, CA, USA). The appropriate populations sampled were identified through distribution records published in Shelley & Sissom [21]. As this scorpion inhabits a variety of habitats across its geographic range, a population can be difficult to define. Based upon previous scorpion dispersal estimates, we designated sampling sites less than 20 km apart as a single historic population [25]. This pooling of samples was conducted in the northern portion of the scorpion’s geographic range where initial analyses indicated little genetic separation among sites. Voucher specimens were deposited in the Zoology collection at Arkansas Tech University. Completely pale *C. pantheriensis* individuals as described by Stahnke [19] were identified from our collections to test the hypothesis of a distinct clade that includes these individuals. For all phylogenetic analyses *C. sculpturatus, C. exilicauda, C. gracilis, C. infamatus, C. suffusus* mitochondrial Cytochrome Oxidase I (COI) sequences were obtained from GenBank for use as outgroups (Table S1). These *Centruroides* species inhabit the northern regions of Mexico along with *C. vittatus* and represent potential sister species to *C. vittatus* [14].

### Molecular Methodology

Total genomic DNA from scorpion pedipalps and a portion of the carapace anterior was extracted with a standard Phenol-Chloroform extraction [27] or with the FastID genomic DNA extraction kit (GeneticIDNA,Inc.). After DNA isolation, each sample was further cleaned by Spermine precipitation to optimize subsequent molecular analyses. Afterwards, the extracted genomic DNA’s were stored in molecular biology grade water (Sigma Chemical Co.) at −20°C until PCR. Initially, the mitochondrial 16S [16] and the nuclear ITS2 region [28] loci were chosen for this study, but then removed as no variation was seen in preliminary DNA sequencing surveys between distant populations. These gene regions appear to discriminate scorpion populations with larger divergence dates but did not discriminate among *C. vittatus* populations [16], [28]–[30]. A 1450-bp portion of the mitochondrial Cytochrome Oxidase I (COI) was amplified with primers described in Folmer et al. [31], Gantenbein & Largiadér [29] and Valdez-Cruz et al. [17]: LCO 1490 (Forward primer) 5'- GGT-CAA-CAA-ATC-ATA-AAG-ATA-TTG-G-3' & COI –N- 2983 (Reverse Primer) 5'-CTT-AAT-AAC-AGC-TAC-AAG-ATG-G-3'. This molecular marker appears well suited for discriminating intraspecific variation among scorpion [5], [6], [9], [10].

In addition, we amplified and sequenced an anonymous nuclear sequence (noncoding genomic locus) from a RAPD investigation for *C. vittatus* microsatellites [32]–[34]. From 24 cloned RAPD fragments, we selected a 728-bp region (Locus 1075) that showed sequence variability in an initial sequence survey among populations. This region was considered a unique locus as it showed no homology with any mitochondrial or nuclear regions in a GenBank BLASTN search. We developed PCR primers to amplify an internal 554 bp region from Locus 1075: Forward 5′-GAA GGG CAG GTT TTC CTG TT-3′ & Reverse 5′-CAT TGC ACA AGT TCG TGA GG-3′. This primer combination only produced amplicons from *C. vittatus*, but not *C. sculpturatus* DNAs.

Each PCR reaction for COI and Locus 1075 was performed in 25-µL aliquots with the following ingredients: 10-µL total genomic DNA, 2X *Taq* Buffer (150 mM Tris-HCl pH 8.5, 40 mM (NH_4_)_2_SO_4_, 3.0 mM MgCl_2_. 0.2% Tween 20 ), 1 mM for each dNTP, 0.5 µM of each primer, 6.25 units *REDTaq* DNA polymerase (Sigma Chemical Co.), 1.6% Dimethyl sulfoxide, 0.6% BSA, and 1.6% Formamide. The cycling conditions consisted of an initial denaturation period of five minutes at 94°C followed with 30 one-minute cycles of 94°C, 50°C annealing, 72°C extension, and a final seven-minute extension at 72°C. After PCR products were verified with agarose electrophoresis in a 0.9% agarose concentration, they were GeneCleaned (Bio 101, Inc.). Forward and reverse DNA sequencing was performed with PCR primers for both sequences at the UAMS DNA Core Sequencing Facility on an Applied Biosystems 3100 Genetic Analyzer, Big Dye Terminator Chemistry, Kit version 1.1 (Foster City, CA, USA). For COI, two additional internal primers along with the previous PCR primers were employed to provide additional sequencing products for a more complete sequence contig: COI-460F 5′-GRG-CYA-YTA-ATT-TTA-TTA-CTA-C-3′ and ScorpNan 5′-CCT-GGC-AAA-ATC-AAA-ATA-TAA-ACC-TC-3′.

After sequencing, all trace files were reviewed by eye and all ambiguous bases removed from further analysis. Alignment of the sequence data was conducted with Clustal X and Geneious Pro 3.7 [35], [36]. After the initial alignment, all COI sequences were converted into their amino acid sequences to verify if any internal stop codons existed. All sequences were deposited in GenBank with the following accession numbers for COI: EF122605–EF122704, EU404114–EU404118, and EU381046–EU381110. GenBank accession numbers for Locus 1075 sequences are: EF122705–EF122787 & JF419172–JF419238. As recombination within mtDNA is reported for scorpions [37], we conducted an analysis for recombination detection with the online version of GARD with default settings [38]. This program can both detect recombination sites and recombinant sequences.

### Phylogenetics

Aligned COI DNA sequences were entered into MODELTEST version 3.7 in HyPhy, and the model of nucleotide sequence evolution (GTR+I+G, -lnL = 7806.20) was chosen with the Akaike (AIC) criteria [38]–[40]. We analyzed these sequences with Maximum Likelihood and Bayesian methods. Maximum likelihood analysis was completed in PAML version 3.14 [41] as it performs phylogenetic analysis with explicit models of nucleotide evolution [41]–[44]. A Neighbor Joining tree was created in PAUP for the likelihood analysis. In addition, 1000 Maximum Likelihood bootstrap repetitions were conducted with the PhyML plugin for Geneious 3.8.5 [45]. Bayesian analyses were conducted with MrBayes 3.1.2 [46] with these parameters: four separate Metropolis-coupled Monte Carlo Markov chains, random starting trees with 20 X 10^6^ generations with samples taken every 100 generations, and 25% of the resultant trees removed as burnin. We produced a 50% majority-rule consensus tree with nodal posterior probability support from the four runs post burn-in. Model parameters for the Bayesian analyses were the same as those in the Maximum likelihood analysis. The average standard of split frequencies was examined to determine if they dropped to a low, convergent value below 0.005. We also reviewed the outputs from the Bayesian analyses with TRACER v1.5 [47] to evaluate the robustness of the Bayesian analyses with respect to burn in, Effective Sample Size, stationary distribution, and posterior.

### Population Statistics

As scorpion population divergence was considered to be potentially minor, additional analyses were conducted to better understand population structure and evolution. Analyses that consider population level processes such as a multitude of haplotypes in populations and recombination encompass parameters that may not be considered in strict phylogenetic analyses [48]–[50]. Haplotype network analysis was conducted on both COI and Locus 1075 sequences in TCS with 90% and 95% connection limits, respectively [48]. We lowered the COI connection limit to 90% as the 95% limit separated individuals from one population into two smaller networks. Any network loops that caused ambiguities were resolved according to Pfenninger & Posada (2002) [51].

To further explore patterns in our data, we conducted several population genetics statistics. These analyses were conducted with Arlequin 3.01 [52]. Populations were grouped into six regional groups based upon clade separations from previous Parsimony, Likelihood, and haplotype network analyses: Northeastern populations (east KS, MO, AR, LA, east TX, and OK); Laredo, TX; Aguirre Springs, NM; central populations combining Trans-Pecos and Central TX (west TX, NM, west KS, CO, and NE); Big Bend National Park, TX; and Hueco Tanks, TX area populations (Hueco Tanks and Chinati Hot Springs). As no clear geographic evidence exists to separate the scorpion populations into geographic regions and mountainous terrain appears to isolate them, we considered the regional groups based upon distinct networks created through the mitochondrial and nuclear haplotype network analyses as robust. In addition, other phylogeographic studies with species in the same geographic region also show similar population structure [53]–[55]. These analyses were conducted with scorpion populations to determine if any evidence of recent expansion and non-neutrality of DNA sequences existed in these regional groups. To test this hypothesis, Fu’s F_s_ and Tajima’s D were calculated in Arlequin 3.01 [52], [56]–[57]. Significant negative values of these statistics indicate non- neutrality and population expansion: Fu’s F_s_ below a p-value of 0.02 indicate population expansion [52], [57].

### Divergence Dating

To further investigate migration and date population separation, coalescent analyses were conducted with Nielsen’s MDIV and *BEAST v.1.6.1. Nielsen’s MDIV was implemented in the Suite of Nucleotide Analysis Programs (SNAP Workbench) [58]–[60]. MDIV has been employed to estimate divergence and migration rates of single genes with a Bayesian model [58], [61]–[63]. MDIV is limited to two models of nucleotide substitution: HKY and Infinite sites. We followed the recommended HKY model for our analysis. COI sequences from selected population pairs were entered with a Hasegawa, Kishino, Yano (HKY) model of nucleotide substitution, 5 X 10^6^ cycles for the Markov chain length, a burn-in time of 5X10^5^ generations, and Mmax and Tmax values of 5 and 10, respectively. In MDIV, M =  migration rate, T = divergence time, and TMRCA = time since the most recent common ancestor. Each population pair was run multiple times, changing the random starting seed each time to produce a more robust analysis. The output from each run was viewed graphically with MS Excel to determine credibility intervals for each population pair (M and Θ, respectively). Population pairs were reanalyzed with higher Mmax or Tmax values if the graphs did not indicate equilibrium in these values. The estimate T_div_ was calculated with the formula T_div_ = TΘ/(2μ). Here Θ and T, the scaled divergence specify Θ and T (time), were estimated with MDIV; a μ value of ∼1% divergence per million years for scorpion mtDNA COI was obtained from rate estimates from the Mediterranean *Mesobuthus* scorpion genus as it represents a robust rate estimate for this mitochondrial gene [29], [64].

The divergence date estimates with the BEAST software were produced with similar parameters to Bayesian analysis done in MrBayes but increased generation time (30X10^6^ generations & 20% burnin) [47], [65]. In these estimates, we reduced the outgroup species to *Centruroides sculpturatus, C. gracilis*, and *C. infamatus.* This analysis estimates several parameters (phylogeny & divergence dates) using a relaxed clock model. We calibrated the clock model with two dates: a Pleistocene divergence (1.5±1 mybp) of the Aguirre Springs, NM population as well as the mean estimate (6,000±2,000 ybp) of the Hypsithermal climatic interval that may have affected the scorpion’s distribution in its Northeastern range limits. Individuals from these populations were constrained into two clades with a normal distribution in the nodes with a Yule tree prior. *C. vittatus* has been introduced into areas outside its geographic range and exists in a wide range of climatic conditions; therefore, tree calibration with geologic or geographic barriers may be inappropriate for this species. The Pleistocene divergence of west Texas populations from those in New Mexico is documented in several species that inhabit this region: velvet ants (*Dilophotopsis concolor*) [66], snakes (*Diadophis punctatus*) [54], tarantulas (*Aphonopelma sp.)* [55], and flightless longhorn cactus beetles (*Moneilema armatum*) [67]. We averaged the Pleistocene divergence dates from these species for our 1.5 mybp estimate. We chose the second calibration date of the Hypsithermal warming interval as it allowed an eastward expansion of many arid adapted species into the Interior Highlands of Missouri, Kansas, and Arkansas approximately 4,000 to 8,000 years before present [68]–[73]. The Hypsithermal interval calibration represents a well-studied eastward expansion period and has been associated with a potentially singular, rapid northeast expansion of species such as *C. vittatus* [68]–[73]. A pattern of recent, rapid expansion of scorpion populations coincident with a Hypsithermal expansion was evident in the phylogenetic analyses with very limited haplotype distribution across the Interior Highlands. In addition, we conducted two other separate analyses in BEAST: an analysis with a singular calibration date at the Hypsithermal expansion (6,000 ybp) and another with no constraints but with the 1% scorpion COI mybp sequence divergence. The first dated run with the two calibration points is considered; however, we include the two additional results as supplementary materials to show variation in our calibration estimates. All Bayesian outputs produced through BEAST were also reviewed in TRACER for robustness in a similar manner to the Mr. Bayes simulations. We summarized the resultant trees in TreeAnnotator v1.6.1 to create a 50% majority-rule consensus maximum clade credibility tree.

### Hypothesis Testing

**Figure 2:**
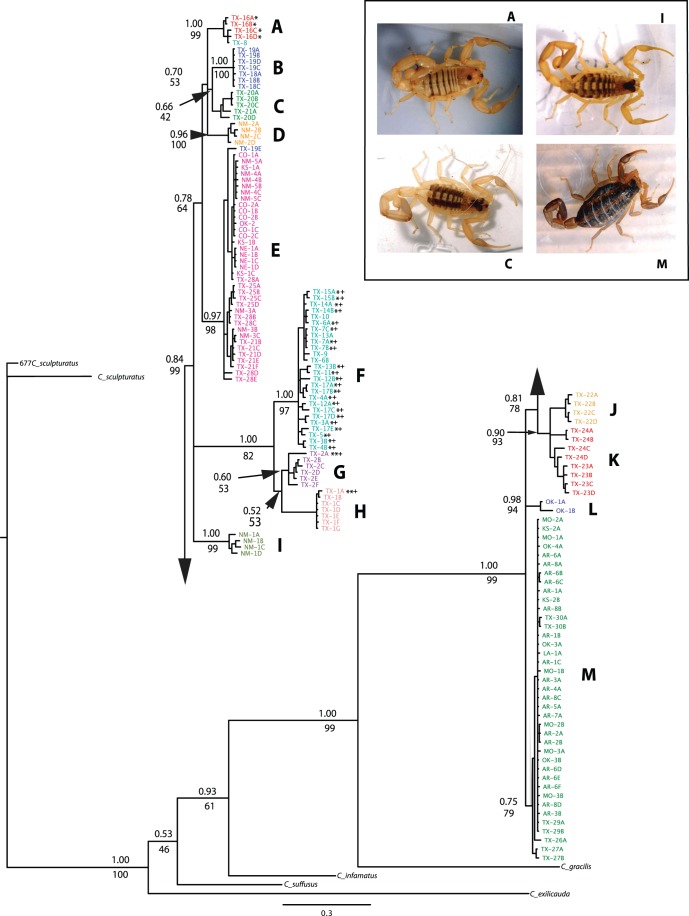
Bayesian Phylogenetic tree for *C. vittatus* populations and outgroups. Numbers above nodes represent Bayesian posterior probabilities: bootstrap support values are shown below. Upper case letter designation of each clade represent networks produced in the TCS haplotype network analysis (see Fig. 4. and results for identification). Asterisked individuals (*) represent those identified as the *C. pantheriensis* variant with double asterisked clades (**) as those clades with all *C. pantheriensis* variants. Individuals or clades marked with a “+” symbol represent those specimens we identified as completely pale forms. Each color represents a general collection region: see results for further details. The inset box shows *C. vittatus* female morphologic diversity in four populations. The population designations are the same as in the Bayesian phylogenetic tree.

We tested several alterative phylogenetic tree topology hypotheses with Bayes factors in the BEAST software through comparing constrained versus unconstrained clades [22]–[24]. First, we tested if any evidence exists for a *C. pantheriensis* clade. This variant was recognized in the Big Bend region and may represent an ecomorph restricted to this region. We identified those individuals that represent *C. pantheriensis* and constrained these samples as a single clade in BEAST. Second, we tested if the two New Mexico populations separated by the Tularosa basin (NM1 & NM2) could exhibit an alternative phylogenetic relationship in the same clade instead of their location in two distant clades in the unconstrained tree (Figure 2). The White Sands formation in the Tularosa Basin has created rapid divergence between lizard taxa in spite of its recent age of 6000 years [74]. Third, we tested if populations in Big Bend National Park could exhibit a tree topology that places them with populations further east in Black Gap WMA (TX16) and Seminole Canyon (TX22). Ecological Niche Modeling analysis suggests optimum environmental contiguity between Big Bend populations and those within 200 km east. Lastly, we tested if *C. vittatus* populations could be placed into Eastern versus Western populations as suggested from a previous allozyme analysis of 15 populations [75]. This work placed *C. vittatus* populations into two distinct clades: a Western clade with those in Big Bend National Park and west from Guadalupe National Monument (generally west of 104 degrees longitude) and a Eastern clade east of the Texas Trans-Pecos region. In all these analyses, a Bayes factor is calculated as twice the –lnL harmonic mean difference between constrained and unconstrained analyses post burnin with differences above 10 suggesting strong evidence for hypothesis rejection [76], [77]. The parameters for these analyses were equivalent to other previous phylogenetic analyses conducted in the BEAST program.

### Morphological Data

We separated individual scorpions into regional population groups delineated from the haplotype network analysis for morphological measurements (see Protocol S1 for a detailed measurement discussion). The scorpions measured included samples from across the range of *C. vittatus* in both the United States and adjacent states of Mexico. We also identified two population groups that consisted of the *C. pantheriensis* variant individuals. Individuals for these analyses were obtained from these museum arthropod collections: the American Museum of Natural History, the California Academy of Sciences, and the Arkansas Tech Museum of Zoology. We measured 356 males and 333 females, then entered each dataset into the NCSS 2007 statistical package (NCSS, Inc.) for Principal Components Analysis (PCA) and Discriminate Function Analysis (DFA) [78]. Although the PCA was able to discriminate among several groups, it could not clearly distinguish population partitions; therefore, we conducted Discriminate Function Analysis (DFA) as it allows prior group prediction then tests the robustness of each individual as a member of the predicted group. Initially, the first three factor residuals for each individual incorporating >75% of the variability from the PCA was entered into the DFA analysis; however, we switched to initial morphometric measurements as they created a better fit between predicted to actual categories. Additionally, we explored base 10 log transformation of the data but the transformations also yielded a lower fit between predicted to actual category. For this statistical analysis, we created 17 male predicted groups and 16 female predicted groups. We also tested the robustness of the DFA through separation of 301 male and female individuals from several random groups.

### Ecological Niche Modeling

We conducted Ecological Niche Modeling in MaxEnt 3.3.3a to determine suitable contemporary habitat and paleoclimatic distributions [79]. The 19 environmental layers were taken from the WorldClim data set (for a 30 arc-second resolution, http://www.worldclim.org) and clipped into ArcGIS 10 (ESRI, Inc.) to encompass the entirety of the *C. vittatus* range [80]. The projected paleoclimatic distribution was produced with a matching climate dataset representing the Last Glacial Maximum (LGM), 21,000 years before present. This data set was created from clipping climate layers available in the Community Climate Model [81]. Both environmental data sets (14 climate layer clips –Protocol S2) and GPS positions for 96 scorpion collection localities were entered into MaxEnt for Ecological Niche Modeling. In the MaxEnt program, we conducted five replicate runs with these parameters: default convergence threshold, maximum iterations (1500), and 25% of the sites for model training [82], [83]. We evaluated the model with the Area Under the Receiving Operating Characteristic (ROC) curve (AUC) that varies from a random prediction of 0.5 to 1 for maximum prediction. We chose 5% as the threshold for the continuous probability produced from the program for suitable climate conditions [82], [83]. The contribution of each climatic variable was assessed through several variables: the percent contribution (the increase in gain of the model for an environmental variable), the permutation importance (the random permutation of a climatic variable to determine the degree the model depends upon the climatic variable), a jacknife of the environmental variables (to determine how well the model operates with only a specific environmental variable, and how well the model operates with the variable omitted and other variables included compared to all environmental variables in the model). The sample sites for the model were determined through field collection localities, museum collection locality data from the morphological data analysis, and collection records from Shelley & Sissom [21]. We identified GPS coordinates for the museum collections from collection locality notes and verifying locations through Google Earth 6.0 (https://earth.google.com). Any collection sites with unidentifiable or uncertain locality data were removed from this analysis.

## Results

### Phylogenetic

We amplified and sequenced 161 COI samples. Each sample produced a sequence of 1450 nucleotides with 111 total haplotypes. The program GARD detected no recombination within the COI or Locus 1075 sequences. The Parsimony analysis with 1451 characters identified 1072 constant characters, 100 uninformative characters, and 279 informative characters. As the parsimony consensus tree reflected Maximum Likelihood and Bayesian trees, and as Maximum Likelihood and Bayesian trees were very similar, only a detailed Bayesian tree is presented (Figure 2). In the Bayesian analyses, Log likelihood values reached a stationary point after 15.82 X 10^6^ generations with an average log likelihood value from the four runs of –8372.15 (Figure 2).

The phylogenetic results identify Trans-Pecos populations as those with the greatest divergence. The Trans-Pecos populations exhibited reciprocal monophyly among all clades except for one individual (TX-19E) being placed into a geographically adjacent clade in central Texas (“E”). The phylogenetic trees show a deep separation between western populations (Chinati HS: “G” and Hueco Tanks: “H”) and Big Bend populations (“F”) and those to the immediate east (i.e., Black Gap: “A”). Interesting, in one Big Bend population, an individual from Persimmon Gap that is geographically adjacent to the Black Gap area (TX-8), clusters with the Black Gap population. A clear division also is seen between Central Texas populations and those to the east (“E” vs. “M”). Another division places Trans-Pecos and Central Texas populations (“A” to “E”) together and is separated from a more eastern/southern clade (“J” to “M”) that contains Laredo and eastern populations. The Aguirre Springs population (“I”) is placed outside the Trans-Pecos clade with robust bootstrap and posterior probability values. The Oliver Lee (“D”) population is contained well within the Trans-Pecos clade. In addition, the separation of the Central Texas clade from the Laredo/Northeastern clade indicates a biogeographic break between populations in south Texas and the northeast from those in western, upland regions.

This species appears to have expanded into their northern and eastern most geographic range boundaries in two distinct patterns. The Nebraska population (NE-1) is placed in the Central Texas clade (“E”) with northern New Mexico, Colorado, and western Kansas populations whereas the eastern Kansas population (KS-2) is placed within the Northeastern clade (“M”). This clade also consists of Interior Highland populations in Oklahoma, eastern Texas, Louisiana, Arkansas, and Missouri.

### Population Statistics

**Figure 3:**
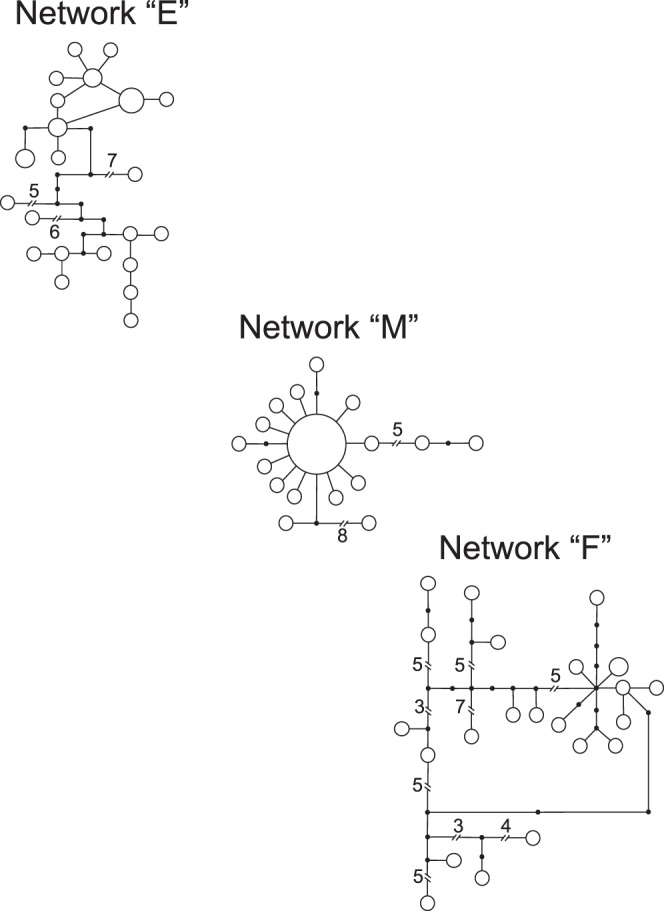
COI TCS networks for the three networks containing several population collection sites. The networks shown here are “E” central Texas/eastern NM/CO/west KS/NE populations, “F” Big Bend, & “M” northeastern populations. The node size represents individual number for each haplotype from singletons (smallest) to six individuals (largest). The large node in network “M” represents 18 individuals. The numbers next to each line break represent mutational steps connecting nodes.

**Figure 4:**
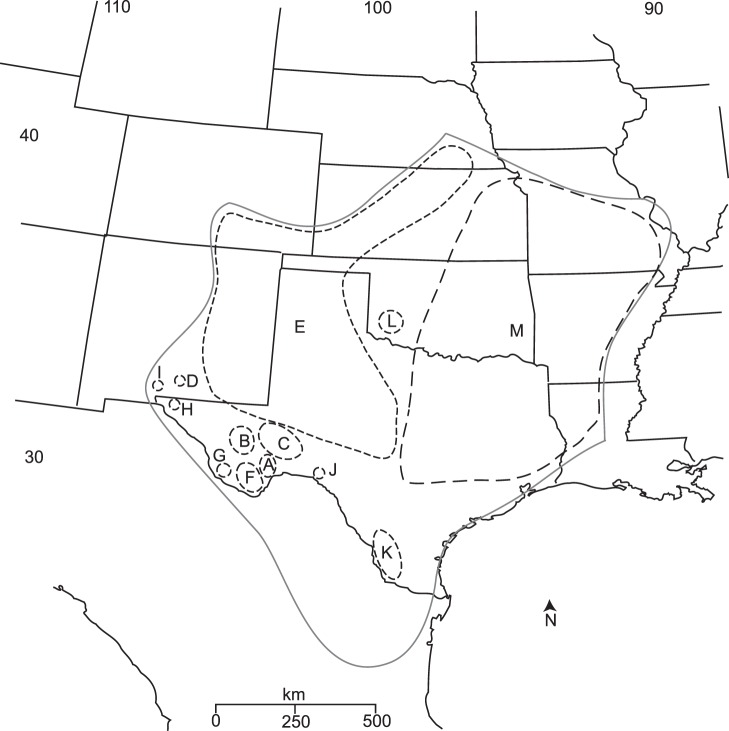
All COI TCS networks overlaid on a geographic map to illustrate network boundaries. These networks are the same as in Figure 2: A: Black Gap, B: Balmorhea Springs/Davis Mts., C: Independence Creek, D: Oliver Lee, E: central Texas/eastern NM/CO/west KS/NE populations, F: Big Bend populations, G: Chinati, H: Hueco Tanks, I: Aguirre Springs, J: Seminole Canyon, K: Laredo/Falcon Lake, L: Wichita Mts., & M: northeastern populations.

**Figure 5:**
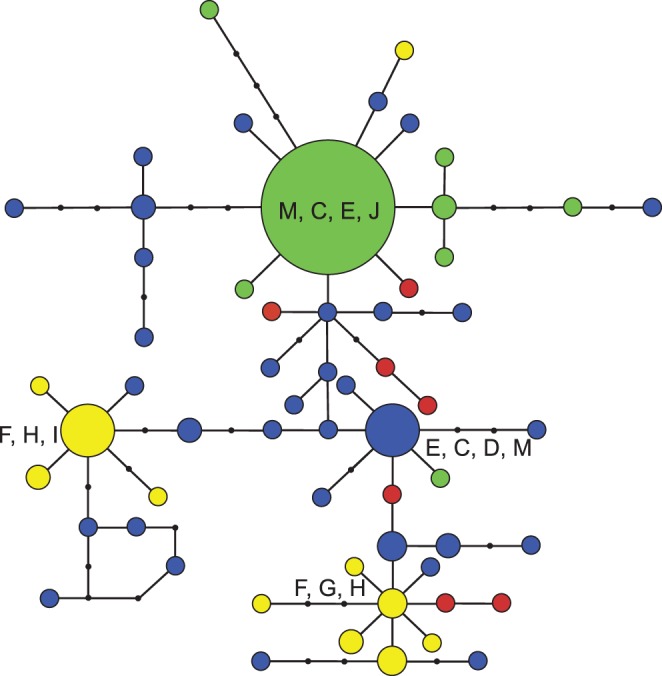
The haplotype network created from the nuclear 1075 locus. Each haplotype node is color coded to the geographic clade with the most represented individuals in the node: Green: northeastern populations, Blue: central Texas/eastern NM/CO/west KS/NE populations, Yellow: Big Bend and Transpecos populations, & Red: Falcon, Seminole Canyon, and Laredo populations. The largest nodes are lettered with all represented COI geographic clades in each node with bold letters representing the most frequent geographic clade. The three largest nodes contained these numbers of individuals: Green: 39, Yellow: 20, & Blue: 15.

In the COI haplotype network analysis, with a 90% confidence limit of 24 steps, 13 networks were created that mirrored clades in the phylogenetic analyses (Figure 2 & Figure 3). We present three COI networks out of the 13 networks created from 106 haplotypes recognized by the TCS analysis; central Texas (“E”), Big Bend (“F”), and the Northeastern region (“M”) (Figure 4.). All other COI networks were restricted to a single population or adjacent populations (e.g., networks A, B, & C; Figure 2 & Figure 4). The COI network with the largest number of haplotypes per sampled individual was Big Bend (“F”) with a maximum of 4 individuals with a single haplotype: conversely, the network with the smallest number of haplotypes per sampled individuals was the Northeastern network (“M”). To summarize, the COI networks show greater genetic variability in the Big Bend region when compared to the populations in the north. The Locus 1075 nuclear marker TCS analysis was calculated with 147 individuals and 544 nucleotides in each sequence. No recombination was detected in this locus. The results are summarized into 60 haplotypes with one large network (Figure 5). In this network, 12 haplotypes were present in multiple individuals with the remaining 48 haplotypes as singletons. To simplify the geographic association in this network, we collapsed the populations into four major geographic regions by color: Northeastern (network “M”-green), central TX (“A, B, C, D, E, & L”-blue), Big Bend (“F, G, H, & I”-yellow), and south TX (“J & K”-red). The Locus 1075 network generally exhibits similar patterns to the COI analyses with three geographic clusters (Northeastern: “M”, Big Bend: “F”, & Central TX: “E”), but exhibits no more than three mutational steps between haplotypes (Figure 5). The small haplotype numbers from the Northeastern populations mirrors the network created with COI and supports the rapid expansion into this region. Haplotypes from the central TX populations were distributed most frequently across the network with south TX haplotypes in three of the four subnetworks. Haplotypes from Northeast and Big Bend regional populations exhibited a strong association to their respective regions with few haplotypes in other subnetwork regions. Lastly, none of the haplotypes associated with the *C. pantheriensis* variant were in a separate sub network, but scattered throughout the Big Bend network.

The population diversity analysis in Arlequin for the COI data showed fewer haplotypes with little separation among haplotypes within the Northeastern region of the geographic range, indicating possible bottlenecks and recent expansion (Table 1). Moreover, Tajima’s D and Fu’s Fs supports the trends presented above, for the COI data. Significant negative values occur in populations in the northeast and Big Bend (Table 1).

**Table 1:** Regional diversity indices for COI sequences.

Regional Populations	Sample &Haplotype #’s ()	Gene Diversity	Nucleotide Diversity	Fu’s Fs	Tajima’s D
Northeastern	46 (24)	0.861+0.049	0.0039+0.0020	−8.90, p = 0.007	−2.247, p = 0.00
Central	60 (36)	0.971+0.0096	0.0180+0.0089	−1.85, p = 0.338	0.011, p = 0.60
Aguirre Springs	4 (4)	1.000+0.177	0.0072+0.0050	0.39, p = 0.374	−0.384, p = 0.51
Laredo	12 (12)	1.000+0.034	0.0156+0.0084	−2.43, p = 0.07	0.175, p = 0.60
Big Bend	26 (22)	0.979+0.0207	0.0071+0.0037	−8.65, p = 0.001	−1.416, p = 0.06
Hueco Tanks	13 (10)	0.923+0.0694	0.0157+0.0083	1.21, p = 0.68	0.895, p = 0.87

Significant values are noted for Northeastern and Big Bend populations for Fu's Fs and Tajima’s D.

### Population Divergence and Hypothesis Testing

**Figure 6:**
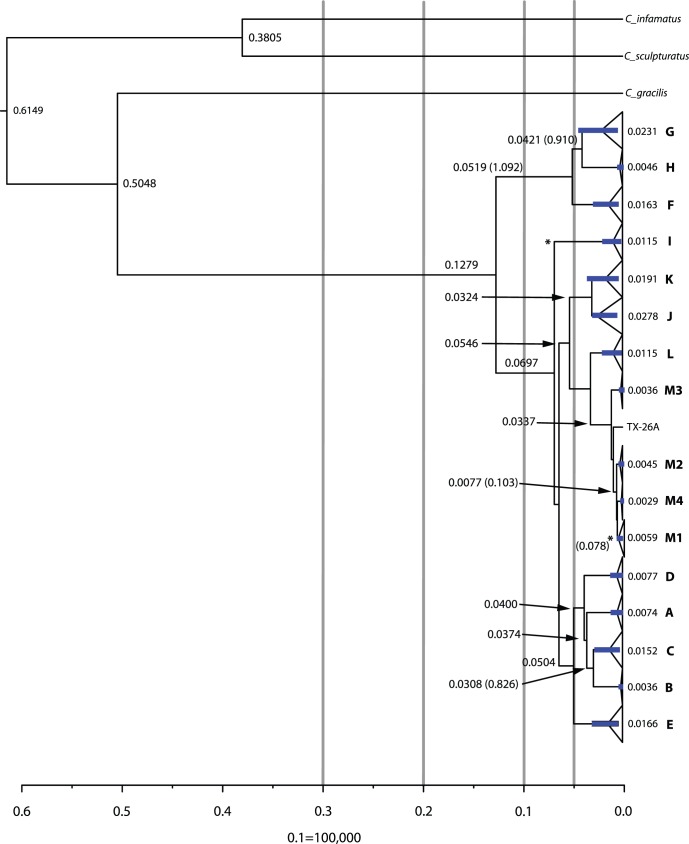
A tree created in BEAST to show regional clade divergence. Letters represent regional network clades as in Figure 4 with northeastern populations further divided into M1 (populations proposed to have been affected through the Hypsithermal expansion), M2 (Louisiana population), M3 (east central Texas population) and M4 (southeast Texas population). Node values represent divergence times in million years with MDIV divergence times in parentheses (see Table S2 for further MDIV divergence statistics and Table S3 for divergence dates calculated in BEAST with different constraints). Error bars (95% confidence intervals) are shown in blue and the calibration points are shown with an asterisk (*).

Both the BEAST and MDIV coalescent analyses support the trend of southwestern populations exhibiting greater divergence than those in the north (Figure 6). Here, we report the results from our first BEAST analyses with the two calibration dates because estimates with a single date near a terminal node may not accurately date deeper divergent nodes [84]. We present estimates from the two other divergence analyses in the Supplementary data section (Table S3). The terminal clades from the BEAST analyses with substantially deeper origin times than the LGM of 21,000 years are populations adjacent to the Rio Grande River (“J & K ”- Laredo, Falcon, & Seminole; Chinati HS- “G”) (Figure 6). The deeper clade divergence times from the calibrated BEAST analysis were markedly shorter than those calculated from the MDIV program, but those for more recent divergence (i.e., Northeastern expansion) fell within the ranges of both methods. MDIV divergence times (T_div_) ranged from 78,000 years for AR/north Texas populations to 900,000 years between the Hueco Tanks/Chinati populations in the southwest. BEAST divergence dates for these populations placed divergence at 7,000 for AR/north Texas to approximately 42,000 ybp for the Hueco Tanks/Chinati populations. All four alternative hypotheses tested with Bayes Factors indicated strong values for rejection (Table 2).

**Table 2:** Bayes factor hypothesis testing for four alternate *C. vittatus* phylogenies.

Constraint	lnL harmonic mean(post burnin)	Bayes Factor	Support for Rejection
Unconstrained Phylogeny	−6786.32	–	–
Hypo 1: support for *C. pantheriensis* clade	−7201.266	829.90	very strong
Hypo 2: single origin of Tularosa NM populations	−6792.47	12.31	very strong
Hypo 3: Single origin in similar ecological niche	−6926.60	280.56	very strong
Hypo 4: Separate origin of western andeastern clades	−7122.50	672.36	very strong

### Morphological Data

We measured 356 males and 333 females for all morphometric analyses (Protocol S1). The PCA scree plot showed all morphometric measurements for both males and females except for pectine teeth number were equally represented in the first PCA factor; pectine teeth number composed the bulk of the second PCA factor. For the Discriminate Function Analyses (DFA), the trial separation of 301 males and females yielded 153/167 (91.2%) males predicted correctly and 130/134 (97.0%) females predicted correctly. For the subsequent DFA analysis, all predicted groups in both male and females were generally placed in actual group categories with greater predicted to actual placement in Trans-Pecos populations (Table 3 & 4). Populations in this region designated as containing *C. pantheriensis* (G & F in Table 3 & 4), exhibited distinct morphometric identities from each other suggesting little evidence beyond color variation, to create a single, unique *C. pantheriensis* designation. Categories representing more northern populations (“E” & “M”) and those in south Texas (“K” & Brownsville), generally exhibited a lower predicted to actual group relationship. We used F values to measure the significant impact of removing characters from the analysis [78]. For males, the three most important variables for separation were movable finger length (removed F- value: 10.82), chela width (removed F- value: 9.64), and chela depth (removed F- value: 8.74). These F values all represent F- probabilities ≤10^−6^. All other characters exhibited removed F-values of <6.50. For females, the three most important variables were the following: chela width (removed F-value: 9.29), carapace length (removed F- value: 6.48), chela depth (removed F-value: 5.72). These F values also all represent F- probabilities ≤10^−6^. All other characters for females exhibited F values of <4.25.

**Table 3:** Morphological classification of female scorpions with Discriminant Function Analysis (DFA) to show correctly classified versus misclassified groups.

Females																	
	Predicted group membership																
Actual group membership	1	2	3	4	5	6	7	8	9	10	11	12	13	14	15	16	Total Actual Groups
1. Brownsville	14	1	2	0	0	2	6	0	0	0	0	0	0	0	0	0	25
2. Tamapulian	1	8	0	0	0	0	0	0	0	0	0	0	0	0	0	0	9
3. Cen TX (E)	2	1	5	2	0	1	2	2	1	2	4	1	2	2	1	0	28
4. Coahuila	0	0	2	34	0	1	0	4	0	0	3	0	0	0	1	0	45
5. CO (E)	1	0	0	0	10	1	1	0	1	0	0	0	2	1	0	0	17
6. NE-N (M)	0	0	0	0	2	3	2	0	0	0	1	0	0	2	0	0	10
7. NE (M)	10	5	1	1	4	4	15	1	0	0	4	0	3	1	0	0	49
8. Laredo (K)	3	3	9	10	1	4	2	12	0	1	2	1	3	1	1	1	54
9. Chinati (G)	0	0	0	0	0	0	0	0	13	0	0	0	0	0	0	1	14
10. Oliver Lee (D)	0	0	0	0	0	0	0	0	0	4	0	0	0	0	0	0	4
11. Big Piney (M)	1	0	0	7	2	1	5	0	0	0	24	0	2	1	0	0	43
12. Falcon Lake (K)	0	0	0	0	0	0	0	1	0	0	0	7	0	0	0	0	8
13. OK (M)	0	0	1	0	0	0	0	1	0	0	1	0	3	1	0	0	7
14. Villanueva (E)	0	0	0	0	0	0	0	0	0	0	0	0	0	3	0	0	3
15. BBend1 (F)	0	0	0	0	0	0	0	0	0	1	0	0	0	0	6	2	9
16. BBend2 (F)	0	0	0	0	0	0	0	0	0	0	0	0	0	0	0	8	8
Total Predicted Groups	32	18	20	54	19	17	33	21	15	8	39	9	15	12	9	12	333

Each row represents actual groups whereas the columns represent predicted groups. Groups identified through the TCS analysis are shown in parentheses. The reduction in classification error (47.5%) shows the accuracy of the DFA compared to random classification. See Protocol S1 for further information concerning population designations and statistical analyses.

**Table 4:** Morphological classification of male scorpions with Discriminant Function Analysis (DFA) to show correctly classified versus misclassified groups.

Males																		
	Predicted group membership																	
Actual group membership	1	2	3	4	5	6	7	8	9	10	11	12	13	14	15	16	17	Total Actual Groups
1. Brownsville	6	2	0	0	1	0	1	2	0	0	0	0	0	0	0	0	1	13
2. Tamapulian	1	11	0	0	0	0	0	0	0	0	0	1	0	0	0	0	0	13
3. Cen TX (E)	0	0	4	0	2	0	2	0	0	2	0	0	0	1	0	0	0	11
4. Coahuila	0	0	1	44	0	0	0	1	0	1	0	1	0	0	0	0	0	48
5. CO (E)	3	1	6	1	15	1	0	1	0	3	0	1	0	1	2	1	0	36
6. NE-N (M)	2	0	0	0	0	5	1	0	0	0	0	2	0	1	2	0	0	13
7. NE (M)	4	0	5	1	3	1	23	1	0	0	0	6	0	2	2	0	0	48
8. Laredo (K)	5	0	2	4	3	1	1	21	1	0	0	3	3	3	0	0	0	47
9. Chinati (G)	0	0	0	0	0	0	0	0	7	0	0	0	0	0	0	0	0	7
10. Ag Sp (I)	0	0	0	0	0	0	0	0	0	4	0	0	0	0	0	0	0	4
11. Oliver Lee (D)	0	0	0	0	0	0	0	0	0	0	4	0	0	0	0	0	0	4
12. Big Piney (M)	2	0	1	0	3	3	4	1	1	0	0	16	0	7	0	0	0	38
13. Falcon Lake (K)	0	0	0	1	0	0	0	1	0	0	0	0	8	0	0	0	0	10
14. OK (M)	0	0	2	0	0	1	1	0	0	0	0	2	0	10	0	0	0	16
15. Villanueva (E)	0	0	0	0	0	0	0	0	0	0	0	0	0	0	9	0	0	9
16. Bbend 1 (F)	0	0	0	1	0	0	0	0	1	0	0	0	0	1	0	12	8	23
17. BBend 2 (F)	0	0	0	0	0	0	0	0	0	0	0	0	0	0	0	1	15	16
Total Predicted Groups	23	14	21	52	27	12	33	28	10	10	4	32	11	26	15	14	24	356

Each row represents actual groups whereas the columns represent predicted groups. Groups identified through the TCS analysis are shown in parentheses. The reduction in classification error (57.6%) shows the accuracy of the DFA compared to random classification. See Protocol S1 for further information concerning population designations and statistical analyses.

### Ecological Niche Modeling

**Figure 7:**
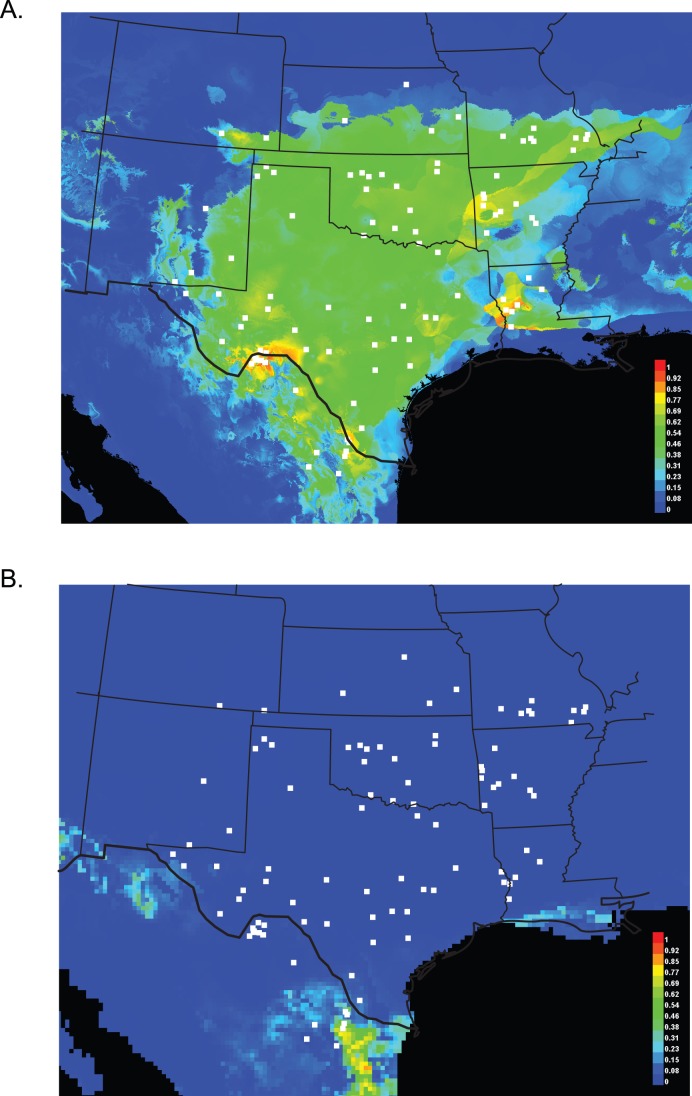
MaxEnt results for *C. vittatus* Ecological Niche Modeling (ENM). Panel A represents current distribution modelling & panel B represents modelling for the Last Glacial Maximum (LGM), approximately 21,000 ybp. Warmer colors (red) represent more optimal climate, whereas cooler color (blue) represent suboptimal climate.

The ecological niche modelling (ENM) analysis shows environmental conditions are favourable for *C. vittatus* across much of its geographic range (Figure 7a). In five separate runs, the average training AUC value from 1500 replicates was 0.885 (sd 0.005; range 9.26–9.38) with the following top predictors: mean temperature of the warmest quarter (18.4%), mean annual temperature (15.6%), mean temperature of the driest quarter (14.1%), temperature seasonality (9.9%), mean temperature of the coldest quarter (9.8%), and precipitation seasonality (7.6%). The jacknife test for variable importance showed ‘mean temperature of the driest quarter’ as the most important variable when used alone. The most optimal conditions for this species exist in the Big Bend region of Texas with a second optimal area further east in Louisiana. Paleoclimatic modelling in MaxENT suggests two separate refugia for this species: one in Chihuahua, Mexico and another in Nuevo León and Tamaulipas, Mexico (Figure 7b).

## Discussion

Our Bayesian phylogenetic tree (Figure 2) shows strong support for the monophly of *C. vittatus* and generally corresponds to the Cuban Buthid phylogeny produced by Teruel et al. [85]. The Bayesian tree indicates strong support for *C. gracilis* as the sister species to *C. vittatus* and support for the *C. exilicauda/C. sculpturatus* separation proposed by Valdez-Cruz et al. [17]. Within *C. vittatus*, all phylogenetic trees showed marked separation among populations in the Trans-Pecos region and other western populations with reduced separation among populations in the northern and eastern regions of the *C. vittatus* geographic range (Figure 2).

*C. pantheriensis* appears to be a morphological variant within *C. vittatus*. The individuals we identified as the *C. pantheriensis* variant are all found in clades centered in the Big Bend region and further west, and are generally associated with the Rio Grande River basin. The Bayes factor hypothesis test provides strong evidence against the existence of a *C. pantheriensis* clade (Table 1). Although superficial evidence exists for a separate *C. pantheriensis* clade, close inspection of the Bayesian tree indicates that within the Big Bend clade (“F”), several individuals exist within the clade that do not correspond to the *C. pantheriensis* variant. In addition, the Black Gap clade (“A”) is geographically adjacent to Big Bend, yet the Bayesian tree places these clades in markedly different branches. These individuals from the Black Gap clade were all initially identified as those belonging to the *C. pantheriensis* variant. However, all the individuals we identified for the phylogenetic analysis as the colorless, pale form of *C. pantheriensis* were those from the Big Bend, Chinati HS, or Hueco Tanks populations. These populations form a separate clade in the *C. vittatus* phylogeny. The nuclear dataset also supports the separation of Big Bend regional populations (Figure 5). Therefore, we cannot exclude the possibility that the colorless, pale form is the result of a unique Big Bend and western Rio Grande basin phylogeographic history and may exhibit incipient speciation.

It is likely that the historic factors that created the Big Bend regional clade produced this unique pale morphological variant along with the other *C. vitttaus* variants observed in this clade, e.g., *C. chisosarius*. Morphometric analysis (DFA, Table 3 & 4) suggests that populations in this region are distinct from each other and neither create a separate Big Bend cluster nor *C. pantheriensis* cluster. This result, when coupled with climate data (ENM), suggests these populations evolve morphological distinction, even in similar environmental conditions. The DFA markedly separates the morphologically similar Aguirre Springs and Oliver Lee populations from each other and also from the pale, colorless individuals in the Hueco Tanks population. These populations also exhibit phylogenetic distinction from each other, yet all are within 100 km. In addition, ENM shows contiguous optimum environmental conditions exist in the Big Bend region and populations further east, yet historic factors appear to over ride contemporary environmental conditions by creating distinct phylogenetic breaks among clades in adjacent populations to the east. This result is noteworthy as the Big Bend region is reported to contain the highest scorpion species diversity in Texas [25]. This high diversity and population isolation is likely a consequence of the region’s topographic complexity [14]. The scorpion fauna in the Big Bend region and adjacent states in Mexico are more likely to show endemism due to scorpion’s low vagility [14], and our results suggest even errant species such as *C. vittatus* evolve marked isolation among populations despite little environmental heterogeneity among populations.

The paleoclimate reconstruction for *C vittatus* shows optimal environmental conditions are predicted to have been restricted to a large area in the southern Rio Grande valley and one further west in Chihuahua, Mexico (Figure 7b). This western and eastern separation is mimicked in other southwestern US desert adapted organisms [83], [86]. However, divergence dating suggests several populations existed prior to the 21,000 LGM date and these scorpions were not restricted to such refugia (Figure 6 & Table S2). The earlier divergence date of several populations also suggests the *C. pantheriensis* variant arose independent of population age and does not appear to be associated with an early divergence date. Although our MDIV estimates are greater than BEAST dates, they are within the estimates for beetle divergence intervals in the same region [67], [87]. MDIV divergence estimates, that include some migration, can indicate deeper divergence than other models that assume no migration between population pairs [58], [88]. Furthermore, MDIV may be more appropriate for species that experienced Pleistocene divergence, i.e., those with finite population ages [88]. It is important to note that credibility intervals created in MDIV analyses show wide ranges; however, we present these results to suggest scorpion populations may have existed in much of their range for many years with historical population divergence and recent expansion into the most northeastern portions of their current geographic range.

### *Centruroides vittatus* Phylogeography and Species Delimitation

The larger phylogeographic analysis of *C. vittatus* suggests many populations expanded after the LGM, but populations along the Rio Grande river existed outside predicted refugia (Figure 6). In addition, distinct phylogeographic breaks occur in the Trans-Pecos and Big Bend regions, as well as within the central Texas and Northeast populations (Figure 4 “E” vs “M”). In this species, the populations along and adjacent to the Rio Grande valley in the western section of the scorpion’s geographic range show the most unique and complex phylogenetic relationship to each other with reciprocal monophyly among populations and separation into markedly distinct clades and haplotype network networks in spite of similar environment and limited geographic distance (Figures 4 & 5). Bayes factor testing (hypothesis 2: single origin of Aguirre Springs and Oliver Lee populations in the Tularosa basin), ENM results, and divergence dating suggests the Tularosa basin was a barrier and these populations independently expanded into these areas after the LGM. The rejection of the third Bayes factor hypothesis (single origin in similar ecological niche of Big Bend area populations) suggests contemporary climate conditions have little effect in homogenizing populations even with optimum environmental conditions. The morphometric data also support the separation of Trans-Pecos populations, as these populations (D, F, G, & I- Table 3 & 4) exhibited the highest predicted to actual numbers. Interestingly, populations south of the Trans-Pecos (Laredo, & Falcon Lake-K & Brownsville) also within the region of a LGM refugium exhibit lower predicted to actual numbers with individuals placed in surrounding and nearby regional populations.

Populations in the Northcentral (“E”) and Northeast (“M”) appear to originate at approximately the date given for the LGM, but the direction of expansion occurred independently into the range extremes (Figure 6). Most northeastern populations in the Interior Highlands of Arkansas, Missouri, and Oklahoma appear to have expanded rapidly coincident with the Hypsithermal expansion of prairie species into this region. Both the mitochondrial COI and nuclear 1075 data support many populations in this region sharing the same haplotype with little genetic separation among populations (Figure 3, 4, 5). This rapid expansion is also verified through the significant Fu’s F and Tajima’s D statistics. No other regional category exhibits significant values for both statistics (Table 1). The morphometric analysis also shows the two northernmost regions (“E” & “M”) exhibit the lowest actual to predicted numbers. When viewed with respect to the ENM modelling, we conclude morphometric similarity among the populations in these regions is due to rapid expansion rather than shared environment. The fourth Bayes factor hypothesis test rejects the division of *C. vittatus* populations into eastern and a Trans-Pecos cluster as proposed in Hedgecock [75]. Our phylogenetic analysis suggests a more complex population subdivision across the *vittatus* geographic range with greater divisions among the Tran-Pecos populations. Lastly, the Wichita Mountain population (“L”) is distinct in all analyses. In the Bayesian tree (Figure 2), it appears as a unique clade: within the BEAST divergence trees (Figure 6.), it appears as a sister clade to the Northeastern population clade (“M”). This population may represent an independent Great Plains expansion, also recovered in the Nightsnake [89].

As seen in other scorpion phylogeographic studies, *C vittatus* exhibits distinct clades that represent isolation and population divergence. Although restricted to a specific geographic region, we find no support for a distinct *C. pantheriensis* clade. However, several populations within the *C. pantheriensis* variant show phylogenetic and morphological distinction and may fall within the parameters for a cohesion species, i.e., populations that exhibit common ancestry, are genetically exchangeable, and ecologically interchangeable [90], [91]. We refrain from delimiting these populations as distinct species as further data from venom analysis and intervening populations are lacking and these data would strengthen any support for further species delimitation [17]. We also stress that a multifactoral approach is important for scorpion species delimitation as scorpion population isolation and speciation appears to be the result of several interwoven factors.

### Conclusions

We conclude the pale *C. pantheriensis* variant is due to *C. vittatus’* unique phylogenetic history in the Big Bend region. In this region, these scorpions exhibit marked phylogenetic and morphological separation despite similar environmental conditions among populations. We show errant scorpions such as *C. vittatus* exhibit a surprising and diverse phylogeographic structure. Our results suggest the Texas Big Bend and Trans-Pecos region house divergent scorpion populations and may represent a region significant for scorpion speciation. This study argues for further phylogeographic research to understand scorpion diversity in this region and the evolution of the morphometric diversity within the *Centruroides* genus. Additional sampling throughout the Trans-Pecos is needed to characterize the diversity and extent of the Big Bend *C. vittatus* clade.

## Supporting Information

Table S1 Population sites, population designations, and GPS coordinates with GenBank accession numbers.
(XLSX)

Table S2 Divergence time estimates from MDIV between selected population pairs.
(XLSX)

Table S3 Divergence dates calculated through three BEAST constraints and MDIV.
(XLSX)

Protocol S1 Morphological data analysis.
(DOC)

Protocol S2 Environmental layers taken from the WorldClim data set and Community Climate Model for Environmental Niche Modeling.
(DOC)
