# Supplementary material for: Species Delimitation and Morphological Divergence in the Scorpion Centruroides vittatus (Say, 1821): Insights from Phylogeography
Source: PLoS One. 2013 Jul 5;8(7):e68282. doi: 10.1371/journal.pone.0068282 (PMC3702564; doi:10.1371/journal.pone.0068282)
Supplement: Protocol S1 — Morphological data analysis. (DOC) [file pone.0068282.s004.doc]

Protocol S1. Morphological data analysis

We obtained *C. vittatus* specimens from the arthropod collections of the American Museum of Natural History, the California Academy of Sciences, and the Arkansas Tech Museum of Zoology for morphological measurements. These specimens included individuals from across the entire range of *C. vittatus*. We measured the following characters: carapace Length, carapace posterior width, chela length, chela width, chela depth, chela movable finger length, metasoma II length, metasoma II width, metasoma V length, metasoma V width, leg IV femur length, right pectine number, and left pectine number. All linear measurements were conducted with a Mitutoyo digital caliper (Model number CD-6) and measured to the 0.01 mm under a Leica MZ-6 dissecting microscope. All individuals were initially measured, then remeasured and checked for error. After all measurements were completed, we remeasured 20 random individuals from all populations to further verify our dataset. For the statistical analyses, we entered the first measurement set. The morphological dataset was base 10 log transformed and entered into the Discriminant Function Analysis (DFA) but the raw measurements were used as the predicted to actual category with the transformed dataset was lower when compared to the untransformed dataset (Females 45.2% - transformed, 47.5%- untransformed: Males 53.3%- transformed, 57.6%-untransformed).

As the samples sizes between males and females among populations varied, we pooled our samples into regional populations determined through the networks created in the TCS analysis. However, in certain instances, we subdivided populations within a region into separate samples for analysis or created smaller samples for analysis. We conducted the DFA in this manner to determine if the NCSS program could discriminate among populations where the phylogenetic analysis indicated a small divergence among populations (NE populations). These populations were subdivided into NE (NE Texas), NE-N (Arkansas), Big Piney (North central AR), & Central Oklahoma. In addition, we separated regional populations into those with smaller samples sizes where the phylogenetic analysis markedly separated these populations from others (i.e., those in the Trans-Pecos area of Texas). The Chinati HS (Table 3: Group #9) and Panther Junction (Table 3: Female Group 16 & Male Group 17) populations were identified as those consisting of the completely pale *C. pantheriensis* variant.

Supplement Table 1. Identified regional populations and sample sizes measured for the morphological analyses (COI TCS Network Id’s are in parentheses):

| Males |  |  | Females |  |
| --- | --- | --- | --- | --- |
|  | N |  |  | N |
| Brownsville, TX | 13 |  | Brownsville, TX | 25 |
| Tamaulipas, Mexico | 13 |  | Tamaulipas, Mexico | 9 |
| Cent TX (E) | 11 |  | Cent TX (E) | 28 |
| Coahuila, Mexico | 48 |  | Coahuila, Mexico | 45 |
| CO, NM (E) | 36 |  | CO, NM (E) | 17 |
| NE-N (M) | 13 |  | NE-N (M) | 10 |
| NE (M) | 48 |  | NE (M) | 49 |
| Laredo, TX (K) | 47 |  | Laredo, TX (K) | 54 |
| Chinati HS, TX (G) | 7 |  | Chinati HS, TX (G) | 14 |
| Las Cruces, NM (I) | 4 |  | Oliver Lee, NM (D) | 4 |
| Oliver Lee, NM (D) | 4 |  | Big Piney, AR (M) | 43 |
| Big Piney, AR (M) | 38 |  | Falcon Lake, TX (K) | 8 |
| Falcon Lake, TX (K) | 10 |  | Oklahoma (M) | 7 |
| Oklahoma (M) | 16 |  | Villanueva, NM (E) | 3 |
| Villanueva, NM (E) | 9 |  | Grapevine, BBendNP (F) | 9 |
| Grapevine, BBendNP (F) | 23 |  | Panther Junction, BBendNP (F) | 8 |
| Panther Junction, BBendNP (F) | 16 |  |  |  |
|  |  |  |  |  |
|  | 356 |  |  | 333 |
