# Supplementary material for: Species Delimitation and Morphological Divergence in the Scorpion Centruroides vittatus (Say, 1821): Insights from Phylogeography
Source: PLoS One. 2013 Jul 5;8(7):e68282. doi: 10.1371/journal.pone.0068282 (PMC3702564; doi:10.1371/journal.pone.0068282)
Supplement: Protocol S2 — Environmental layers taken from the WorldClim data set and Community Climate Model for Environmental Niche Modeling. (DOC) [file pone.0068282.s005.doc]

Protocol S2. Environmental layers taken from the WorldClim data set and Community Climate Model for Environmental Niche Modeling.

**BIOCLIM 1:** Mean Annual Temperature

**BIOCLIM 4:** Temperature seasonality (standard deviation *100)

**BIOCLIM 8:** Mean temperature of the wettest quarter

**BIOCLIM 9:** Mean temperature of the driest

**BIOCLIM 10:** Mean temperature of the warmest quarter

**BIOCLIM 11:** Mean temperature of the coldest quarter

**BIOCLIM 12:** Annual precipitation

**BIOCLIM 13:** Precipitation of the wettest period

**BIOCLIM 14:** Precipitation of the driest period

**BIOCLIM 15:** Precipitation seasonality (Coefficient of Variation)

**BIOCLIM 16:** Precipitation of the wettest quarter

**BIOCLIM 17:** Precipitation of the driest quarter

**BIOCLIM 18:** Precipitation of the warmest quarter

**BIOCLIM 19:** Precipitation of the coldest quarter
